# Effect of cervical changes on the cesarean scar topography after primary cesarean sections: an observational study

**DOI:** 10.1186/s12884-025-08396-0

**Published:** 2025-12-01

**Authors:** Ahmed G. Elnajar, Ramy Arm, Rehab M. AbdelRahman, Amr Ahmed Mahmoud Riad, Joseph Hozain, Mostafa Mohamed Othman Helal

**Affiliations:** Obstetrics and Gynecology Department, Faculty of Medicine, Ain Shams University, Cairo, Egypt

**Keywords:** Cesarean Scar Topography, Cesarean Sections, Cesarean scar defect, Niche, Delivery, Myometrial thickness, Isthmocele, Uterine dehiscence, Uterine diverticulum

## Abstract

**Background:**

The increasing global rate of cesarean sections (CS) has raised concern about associated long-term complications, particularly uterine scar niches. A niche represents a localized myometrial defect at the hysterotomy site, which may lead to abnormal bleeding, pelvic pain, or subfertility.

**Objective:**

to assess the scar area, niche formation and isolated myometrial defect creation after CS in women undergoing their 1st CS at various stages of 1st stage of labor, irrespective of gestational age.

**Methods:**

This prospective observational cohort study was conducted at Ain Shams University Maternity Hospital between April 2024 and February 2025 and included 92 women who underwent their first CS for different obstetric indications at various stages of 1st stage of labor. Cervical changes were recorded just before the cesarean section, then all patients were recalled for TVUS three months postoperatively to evaluate scar location, the presence of a niche or isolated myometrial defect, and residual and total myometrial thickness.

**Results:**

Among the 92 enrolled women, scar-related abnormalities were closely linked to cervical and fetal labor characteristics rather than general maternal or operative factors.

While maternal age, BMI, gestational age, fetal weight, operation time, and blood loss showed no significant differences between women with and without niches or isolated myometrial defects (*p* > 0.05 for all comparisons), cervical dilation (6.0 ± 2.3 cm vs. 4.1 ± 1.9 cm), effacement (58.2 ± 24.4% vs. 36.6 ± 24.4%), and advanced fetal station (0 or + 1 in 67.6% of niche cases) were significantly associated with niche formation (*p* < 0.001 for all).

Niche width and depth correlated positively with cervical dilation (*r* = 0.591, 0.385; respectively), effacement (*r* = 0.547, 0.381; respectively), and fetal descent (*r *= 0.766. 0.545; respectively) (*p* < 0.05 for all), Conversely, isolated myometrial defect were more frequent in CS performed at less progressive cervical changes and higher fetal head station.

Anatomically, niches were more frequent in isthmic scars (55.9%), whereas isolated defects predominated in corpus scars (52.0%).

Collectively, these findings suggest that advanced cervical changes contribute to wider and deeper niches, whereas insufficient cervical remodeling favors isolated defects development.

**Conclusion:**

Advanced cervical changes contribute to wider and deeper niches, whereas insufficient cervical remolding favors isolated defect development. Therefore, labor dynamics represent a pivotal determinant of cesarean scar healing and postoperative uterine integrity.

## Introduction

A uterine niche is defined as a pouch-like defect that develops at the site of a previous cesarean section scar due to impaired myometrial healing. It has also been described in the literature using alternative terms, including uterine isthmocele, cesarean scar defect, uterine dehiscence, and diverticulum. On imaging, a niche typically appears as a triangular hypoechoic or anechoic area at the scar site, or as an indentation of at least 2 mm within the myometrium [1]. An isolated myometrial defect refers to a localized area of thinning or discontinuity in the uterine myometrium at the site of a previous cesarean section scar. Unlike a niche, this defect does not communicate with the uterine cavity and is confined to the myometrium. These variations in scar healing highlight the complexity of post-cesarean uterine remodeling and the need to better understand factors that contribute to scar abnormalities [2].

Over the past decade in Egypt, there has been a notable increase in cesarean section (CS) deliveries. The most recent data from the Egypt Demographic and Health Survey (EDHS) indicate a CS rate of 52%, implying potential overutilization of cesarean delivery or its application for non-indicated clinical reasons [3]. Given the rising frequency of CS, the clinical burden of scar-related complications is expected to grow. Indeed, the prevalence of uterine niche following cesarean section, as evaluated by sonohysterography, has been reported to range between 56% and 84% [4], underscoring the high likelihood of encountering these defects in clinical practice.

The occurrence of cesarean uterine scar defects is not only rising but is also associated with long-term complications. Among the factors contributing to the development of cesarean section defects or niches, surgical techniques employed for uterine incision closure have been identified as critical determinants [5]. However, surgical practice alone does not fully explain the variability in scar outcomes, suggesting that obstetric factors also play a pivotal role.

The European Niche Taskforce has standardized the definition, describing a niche as an indentation of at least 2 mm within the myometrium at the cesarean scar site, usually assessed using transvaginal ultrasound [6]. Obstetric factors such as advanced cervical dilatation (> 5 cm), prolonged labor (> 5 h), and low fetal station at the time of cesarean delivery have also been associated with the formation of large defects. This is thought to be due to thinner or less vascularized myometrial tissue, which adversely affects healing capacity [1]. These findings emphasize that both surgical and obstetric parameters must be considered when studying cesarean scar healing.

The presence of a niche has been linked to various clinical manifestations, including abnormal uterine bleeding (AUB), such as prolonged menstruation and postmenstrual spotting, which occurs in approximately 30% of affected women. Additional symptoms commonly reported include dysmenorrhea and chronic pelvic pain. Moreover, the accumulation of blood, mucus, and fluid within the niche, cervix, and uterus is hypothesized to contribute to secondary subfertility by impeding sperm penetration or embryo implantation [7]. Surgical repair of the niche may benefit patients experiencing subfertility and postmenstrual spotting. However, in the absence of associated symptoms, the presence of a niche may be clinically insignificant [8]. Collectively, these considerations highlight the importance of identifying risk factors and mechanisms underlying niche and defect formation, which formed the rationale for the present study.

Primary Objective: to assess the scar area, niche formation and isolated myometrial defect creation after cesarean section in women who will have their 1 st cesarean section at various stages of 1 st stage of labor.

Secondary Objective: to study potential Risk factors for the development of a niche & isolated myometrial defect.

## Patients and methods

### Study design

This prospective observational cohort study was conducted at Ain Shams University Maternity Hospital between April 2024 and February 2025. It included women undergoing their first cesarean section for different obstetric indications at various stages of 1 st stage of labor. Participants were monitored to evaluate postoperative uterine changes, with a focus on the relationship between intra-labor factors and scar-related complications.

Among the included women, 16 of them delivered near term (< 37 weeks) (of whom 11 were at 36 weeks and 5 at 35 weeks of gestation).

#### Inclusion criteria

Eligible participants were women undergoing their first cesarean section after 28 weeks of gestation and gave informed consent to participate.

#### Exclusion criteria

Women were excluded if they had a history of previous uterine surgery or cesarean section (to avoid confounding scar morphology), refused participation(patient`s right), had twin pregnancies (due to uterine overdistension Which can affect the study results), diabetes mellitus, smokers, used immunosuppressive drugs, or had uterine anomalies or fibroids (due to their effects on uterine anatomy and healing). Women who developed postoperative complications (e.g., fever, Intra-peritoneal sepsis, anemia) were withdrawn from the statistical analysis and replaced to ensure the integrity of the healing assessment.

### Sampling method

Cases were identified and recruited at Ain Shams University Maternity Hospital. Written informed consent was obtained from all participants prior to data collection.

### Sample size

The Sample size was calculated using the PASS 15 software. Setting the confidence level at 95% and the margin of error at 10%, a sample of 90 women was needed to detect an expected niche formation incidence of 34.4% based on *Dogru *et al*.* [9]. Allowing for a 10% dropout rate, the final sample included 100 women.

### Ethical considerations

The study protocol was reviewed and approved by the Ethical Committee of the Faculty of Medicine, Ain Shams University. All participants received a full explanation of the study procedures and provided written informed consent.

### Study interventions

All patients presenting in labor at Ain Shams University Maternity Hospital and indicated for cesarean delivery were invited to participate. Each patient was scheduled for a follow-up visit at 3 months postpartum. The following assessments were performed:

#### History

Data were collected on personal details (age, name, special habits), and medical history (chronic diseases such as diabetes, hypertension, cardiac, pulmonary, hepatic, renal, autoimmune conditions), allergies, current medications, previous surgeries, and obstetric history (gravidity, parity, gestational age, labor duration, premature rupture of membranes, oxytocin use, and cesarean indication).

#### Clinical examinations

A full physical examination was conducted just preoperatively. Cervical dilatation was assessed before the cesarean section. Additional factors potentially affecting wound healing such as steroid use during pregnancy, pre-eclampsia, infections, intraoperative complications, and maternal BMI were also evaluated.

#### Laboratory investigations

A complete blood count (CBC) was obtained prior and after delivery.

#### CS operation

All cesarean deliveries were performed by surgeons of the same rank. Postoperative complications were documented, and patients with complications (e.g., endometritis, intra-peritoneal infection) were excluded from final analysis. All uteruses were continuously fixed in two layers The parietal peritoneal repair was done. All patients received 30 IU oxytocin infusion postoperative.

#### Radiological Assessment

**Fig. 1:**
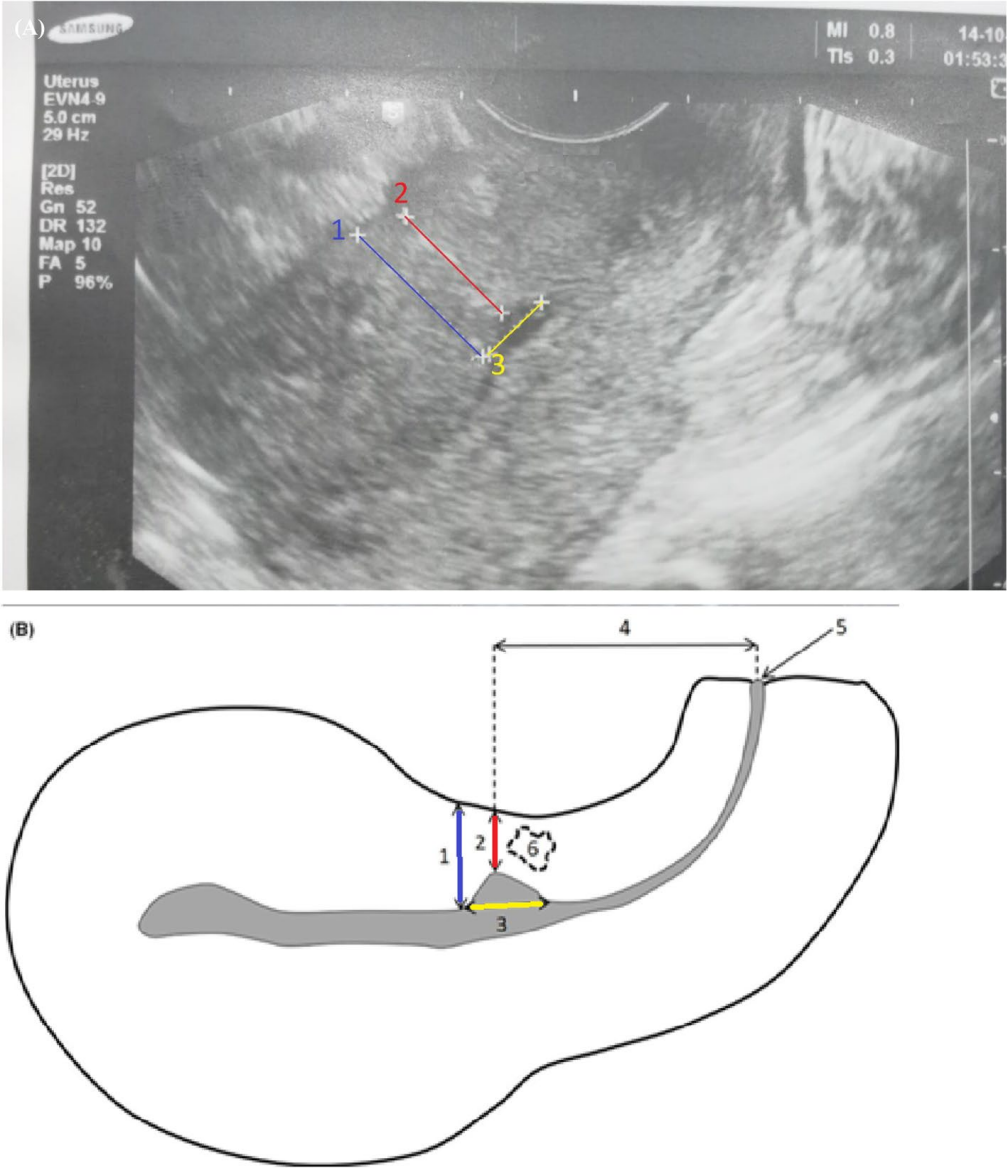
**A** A patient`s Transvaginal ultrasound showing the assessment of (1)the total myometrial thickness (Blue line) (2) the residual myometrial thickness (Red Line) (3) the niche width (the yallow line). **B** A schematic illustration showing the position of the cesarean section (CS) scar and the assessed parameters: (1) total myometrial thickness(Blue line), (2) residual myometrial thickness(Red Line), (3) scar defect width(Yallow Line), (4) distance between the scar and the external cervical os, (5) external cervical os, and (6) isolated myometrial defect width

Preoperative trans-abdominal ultrasounds was performed and 3 months postpartum patients recalled, trans-vaginal ultrasonography was conducted by a single experienced sonographer (> 10 years in obstetric imaging) using a WS80A ELITE Samsung Ultrasound Machine. The following measurements were taken: total myometrial thickness, residual myometrial thickness (RMT), scar location, scar width, and the distance between the scar and the external cervical os. Scar defects, whether contacting the uterine cavity or not (isolated myometrial defect), and the presence and size of niches were recorded (Fig. 1).

#### Study outcomes

##### Primary Outcome

Presence of scar niche formation following cesarean section.

##### Secondary Outcomes

Total myometrial thickness, residual myometrial thickness (RMT), presence of isolated myometrial defects, distance between the scar and the external OS, scar location, and scar width.

### Statistical methods

**Fig. 2:**
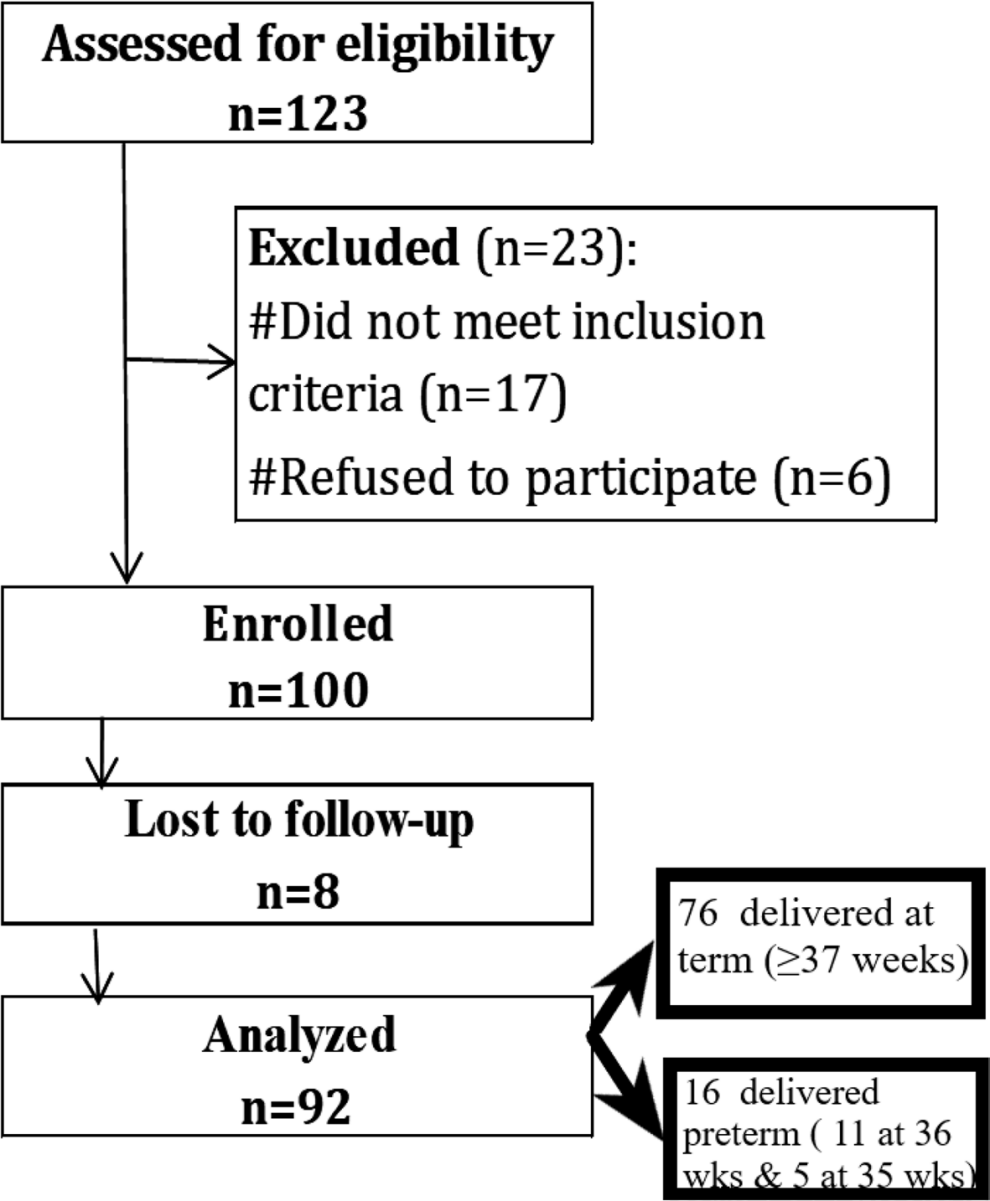
Study flow diagram. (the 92 participants divided into 76 women (82.6%) delivered at term (≥ 37 weeks), whereas 16 women (17.4%) delivered near term (< 37 weeks). All preterm cases were near-term, including 11 women around 36 weeks and 5 women around 35 weeks of gestation

The collected data were coded, tabulated, and statistically analyzed using IBM SPSS statistics (Statistical Package for Social Sciences) software version 28.0, IBM Corp., Chicago, USA, 2021. Quantitative data tested for normality using Shapiro–Wilk test, then described as mean ± SD (standard deviation) as well as minimum and maximum of the range, and then compared using independent t-test. Qualitative data described as number and percentage and then compared using Chi square test and Fisher’s Exact test. Correlations were tested by Pearson correlation test and Spearman correlation test. Bonferroni test was used for post hoc comparisons. The level of significance was taken at p-value ≤ 0.050 was significant, otherwise was non-significant (Fig. 2).

## Results

Table 1 demonstrates that there were no statistically significant differences between the niche and no-niche groups with respect to maternal age, BMI, gestational age, fetal weight, operative duration, blood loss, or fetal presentation (*p* > 0.05 for all comparisons). These findings suggest that baseline demographic and intraoperative characteristics may not play a decisive role in the development of uterine niches.

**Table 1:** Comparison according to niche regarding demographic and operative characteristics

Characteristics		Niche (Total = 34)	No niche (Total = 58)	p-value
**Maternal age (years)**		28.0 ± 3.1	27.3 ± 2.8	^0.283
**Maternal BMI (kg/m**^**2**^**)**		29.3 ± 2.9	28.3 ± 3.0	^0.134
**Gestational age (weeks)**		37.7 ± 1.5	37.9 ± 1.3	^0.482
**Fetal weight (gm)**		2884.9 ± 365.1	2925.8 ± 310.2	^0.569
**Operation duration (minutes)**		56.9 ± 8.1	58.1 ± 8.3	^0.482
**Blood loss (mL)**		545.8 ± 96.2	562.8 ± 88.7	^0.392
**Fetal presentation**	**Head**	28 (82.4%)	53 (91.4%)	§0.318
	**Breach**	6 (17.6%)	5 (8.6%)	

*BMI* Body mass index

^Independent t-test

§Fisher’s Exact test

Table 2 reveals significant associations between cervical characteristics and the presence of a niche. Cases with a niche demonstrated significantly lower fetal station (e.g.,Station 0 or + 1) (*p* < 0.001), as well as greater cervical dilatation and effacement compared to cases without a niche (*p* < 0.001 for both). These findings indicate that cervical changes prior to cesarean delivery may contribute to structural alterations in scar healing.

**Table 2:** Comparison according to niche regarding cervix characteristics

Characteristics		Niche (Total = 34)	No niche (Total = 58)	p-value
**Station**	**−3**	2 (5.9%)a	10 (17.2%)a	§ < 0.001*
	**−2**	4 (11.8%)a	19 (32.8%)b	
	**−1**	5 (14.7%)a	23 (39.7%)b	
	**0**	10 (29.4%)a	3 (5.2%)b	
	**1**	13 (38.2%)a	3 (5.2%)b	
**Dilatation (cm)**		6.0 ± 2.3	4.1 ± 1.9	^ < 0.001*
**Effacement (%)**		58.2 ± 24.4	36.6 ± 24.4	^ < 0.001*

^Independent t-test

^#^Chi square test, homogenous groups had the same symbol "a and b" based on post hoc Bonferroni test

^*^Significant

Table 3 indicates that cases with a niche were significantly more likely to have scars located at the isthmus (*p* = 0.006) and less likely to have scars confined to the cervical canal. This highlights the anatomical predisposition of the isthmic region to post-cesarean scar abnormalities.

**Table 3:** Comparison according to niche regarding scar site

Characteristics		Niche (Total = 34)	No niche (Total = 58)	p-value
**Site**	**Corpus**	12 (35.3%)a	15 (25.9%)a	#0.006*
	**Isthmus**	19 (55.9%)a	20 (34.5%)b	
	**Cervical canal**	3 (8.8%)a	23 (39.7%)b	

^#^Chi square test, homogenous groups had the same symbol "a and b" based on post hoc Bonferroni test

^*^Significant

Table 4 demonstrates that demographic and operative variables—including maternal age, BMI, gestational age, fetal weight, operative duration, blood loss, and fetal presentation—did not differ significantly between the group with isolated myometrial defect and the group without isolated myometrial defect (*p* > 0.05 for all). These results suggest that factors beyond baseline demographics and operative conditions are likely to influence the development of isolated myometrial defects.

**Table 4:** Comparison according to isolated myometrial defect regarding demographic and operative characteristics

Characteristics		Isolated Myom. Defect (Total = 25)	No Isolated Myom. defect (Total = 67)	p-value
**Maternal age (years)**		27.7 ± 3.1	27.5 ± 2.8	^0.834
**Maternal BMI (kg/m**^**2**^**)**		28.9 ± 3.5	28.6 ± 2.9	^0.733
**Gestational age (weeks)**		38.1 ± 1.5	37.7 ± 1.3	^0.298
**Fetal weight (gm)**		2977.6 ± 328.9	2885.7 ± 329.6	^0.237
**Operation duration (minutes)**		59.9 ± 9.1	56.8 ± 7.7	^0.109
**Blood loss (mL)**		542.6 ± 109.7	561.7 ± 84.0	^0.377
**Fetal presentation**	**Head**	21 (84.0%)	60 (89.6%)	§0.482
	**Breach**	4 (16.0%)	7 (10.4%)	

*BMI* Body mass index

^Independent t-test

^§^Fisher’s Exact test

Table 5 shows that cases with isolated myometrial defects presented significantly Higher fetal head stations (e.g. Station −3,−2) (*p* = 0.005), as well as reduced cervical dilatation and effacement (p < 0.001 for both), compared with cases without defects. These findings suggest that minimal cervical changes prior to cesarean may predispose to poor scar remodeling and defect formation.

**Table 5:** Comparison according to isolated myometrial defect regarding cervix characteristics

Characteristics		Isolated Myom. Defect (Total = 25)	No isolated myom. defect (Total = 67)	p-value
**Station**	**−3**	7 (28.0%)	5 (7.5%)	§0.005*
	**−2**	8 (32.0%)	15 (22.4%)	
	**−1**	6 (24.0%)	22 (32.8%)	
	**0**	4 (16.0%)	9 (13.4%)	
	**1**	0 (0.0%)	16 (23.9%)	
**Dilatation (cm)**		3.6 ± 1.4	5.3 ± 2.3	^0.001*
**Effacement (%)**		29.4 ± 21.2	50.2 ± 26.1	^0.001*

^Independent t-test

^§^Fisher’s Exact test, homogenous groups had the same symbol "a and b" based on post hoc Bonferroni test

^*^Significant

Table 6 indicates that isolated myometrial defects were significantly more frequent in scars located at the corpus (*p* = 0.001) compared with the cervical canal, which was less commonly involved. This anatomical association may reflect differences in myometrial structure and vascular supply across uterine segments.

**Table 6:** Comparison according to isolated myometrial defect regarding scar site

Characteristics		Iso.Myo.Defect (Total = 25)	No Iso.Myo. defect (Total = 67)	p-value
**Site**	**Corpus**	13 (52.0%)a	14 (20.9%)b	#0.001*
	**Isthmus**	11 (44.0%)a	28 (41.8%)a	
	**Cervical canal**	1 (4.0%)a	25 (37.3%)b	

^#^Chi square test, homogenous groups had the same symbol "a and b" based on post hoc Bonferroni test

^*^Significant

Table 7 demonstrates that the mean myometrial thickness among the studied cases was 10.9 ± 2.0 mm, ranging from 6.0 to 17.0 mm. The incidence of niches and defects was 37.0% and 27.2%, respectively. These prevalence rates are consistent with previous studies reporting substantial rates of cesarean scar abnormalities, underscoring their clinical relevance.

**Table 7:** Uterine findings of the studied cases

Characteristics	Mean ± SD	Range
**Total myometrial thickness (mm)**	10.9 ± 2.0	6.0–17.0
	**N**	**%**
**Niche**	34	37.0%
**Isolated myometrial defect**	25	27.2%

Total = 92

Table 8 highlights that both niche width and depth showed significant positive correlations with fetal station, cervical dilatation, and effacement (*p* < 0.05 for all). Conversely, residual myometrial thickness was negatively correlated with these cervical parameters (*p* < 0.05). These results suggest that advanced cervical changes may contribute to wider and deeper niches, potentially compromising scar integrity.

**Table 8:** Correlations of niche dimensions

**Characteristics**		**Niche width**	**Niche depth**	**Residual**
**Maternal age (years)**	**r**	0.026	−0.065	−0.529
	**^*p*-value**	0.884	0.715	0.001
**Maternal BMI (kg/m**^**2**^**)**	**r**	−0.075	−0.148	−0.008
	**^*p*-value**	0.671	0.403	0.963
**Gestational age (weeks)**	**r**	−0.183	−0.066	0.128
	**^*p*-value**	0.299	0.710	0.471
**Fetal weight (gm)**	**r**	−0.247	−0.132	0.170
	**^*p*-value**	0.159	0.457	0.338
**Operation duration (minutes)**	**r**	−0.203	−0.212	−0.027
	**^*p*-value**	0.249	0.229	0.878
**Blood loss (mL)**	**r**	−0.040	0.007	−0.266
	**^*p*-value**	0.822	0.970	0.129
**Station**	**r**	0.766	0.545	−0.381
	**#*p*-value**	< 0.001*	0.001*	0.026*
**Cervical dilatation (cm)**	**r**	0.591	0.385	−0.368
	**^*p*-value**	< 0.001*	0.024*	0.032*
**Cervical effacement (%)**	**r**	0.547	0.381	−0.403
	**^*p*-value**	0.001*	0.026*	0.018*

Total = 34

*BMI* Body mass index

^Pearson correlation test

^#^Spearman correlation test

^*^Significant

Table 9 demonstrates that the width of isolated myometrial defects was negatively correlated with fetal station, cervical dilatation, and effacement (p < 0.05 for all). These findings imply that isolated Myometrial defects may form in settings of minimal cervical change, contrasting with niches that are associated with greater cervical involvement.

**Table 9:** Correlations of isolated myometrial defect dimensions

**Characteristics**		**Isolated Myom. Defect width**
**Maternal age (years)**	**r**	0.053
	**^*p*-value**	0.801
**Maternal BMI (kg/m**^**2**^**)**	**r**	0.137
	**^*p*-value**	0.513
**Gestational age (weeks)**	**r**	−0.158
	**^*p*-value**	0.451
**Fetal weight (gm)**	**r**	−0.185
	**^*p*-value**	0.375
**Operation duration (minutes)**	**r**	−0.275
	**^*p*-value**	0.184
**Blood loss (mL)**	**r**	0.306
	**^*p*-value**	0.137
**Station**	**r**	−0.491
	**#*p*-value**	0.013*
**Cervical dilatation (cm)**	**r**	−0.447
	**^*p*-value**	0.025*
**Cervical effacement (%)**	**r**	−0.526
	**^*p*-value**	0.007*

Total = 25

*BMI* Body mass index

^Pearson correlation test

^#^Spearman correlation test

^*^Significant

Table 10 demonstrates that After analysis of different demographic and clinical characteristics, station was a significant independent factor that its positive directions increased the likelihood of niche formation, while effeacement decreased the likelihood of niche formation. Regarding defect, corpus site was a significant independent factor that increased the likelihood of defect formation, while effeacement decreased the likelihood of defect formation.

**Table 10:** Logistic regression for independent factors affecting niche and defect formation

Factors	β	SE	p-value	Odds ratio (95% CI)
Niche				
Station	2.801	0.736	< 0.001*	16.468 (3.893–69.668)
Effacement	−0.087	0.032	0.007*	0.917 (0.861–0.977)
Constant	5.936	2.105	0.005*	
Isolated Myometrial Defect				
Corpus site	1.926	0.608	0.002*	6.862 (2.083–22.607)
Effacement	−0.046	0.014	0.001*	0.955 (0.930–0.981)
Constant	0.079	0.501	0.874	1.083

*β* Regression coefficient, *SE* Standard error, *CI* Confidence Interval

^*^Significant

## Discussion

This prospective study, which was conducted at Ain Shams University Maternity Hospital and included 92 women, investigated the influence of cervical changes during labor on cesarean scar healing and topography, with particular focus on the development of uterine niches and isolated myometrial defects. The findings demonstrated that advanced cervical dilation, effacement, and fetal descent were significantly associated with wider and deeper niches, whereas minimal cervical remodeling and higher fetal stations were linked to isolated myometrial defects. These results highlight that intrapartum cervical dynamics represent key determinants of postoperative scar integrity.

Our results are consistent with previous literature examining the frequency and determinants of post-cesarean scar defects. The prevalence of niche formation in our cohort (37%) aligns with the wide range reported in literature, as noted by *Armstrong *et al*.,* [10]. Similarly, *Dogru *et al*.* [9] documented an overall niche prevalence of 34.2%, with large niches occurring in 17.1% of cases. while *Galal *et al*.* [11] and *Elkashef *et al*.* [12] documented 18.7%, and 77.2% respectively. These discrepancies likely reflect differences in diagnostic thresholds, methodology, and patient populations. Our mid-range prevalence suggests that niche formation is relatively common but may vary according to diagnostic criteria and study design. This supports the representativeness and external validity of our cohort, while the single-center design remains a limitation.

In the present study, no statistically significant differences were observed in maternal age, BMI, gestational age, fetal weight, fetal presentation, operative time, or blood loss between women with or without a niche or isolated myometrial defect. This indicates that niche formation in our cohort was mainly influenced by intrapartum rather than demographic factors, emphasizing the importance of labor dynamics as primary contributors. These findings are consistent with those of *Lumbanraja *et al*.* [13]*,* and *Savukyne *et al*.* [14], who found no significant association between niche development and maternal characteristics (*p* > 0.05), (*p* = 0.049) respectivly. In contrast, *Elkashef *et al*.* [12] reported higher age (29.43 ± 5.37 vs. 27.19 ± 6.93 years) and BMI (with a mean BMI of 27.15 ± 4.17 in the defect group vs. 25.28 ± 2.90 in the control group) among women with defects, possibly due to population differences or sample size effects. Also *Tang *et al*. *[15] reported that factors such as intraoperative blood loss, and CS duration were significantly associated with cesarean scar defects, whereas maternal age, parity, and anesthesia type showed no association. Collectively, these comparisons suggest that while demographic factors may play a minor role, cervical and intrapartum conditions appear to be stronger determinants of scar outcomes.

Evaluation of the cervical characteristics in the current study demonstrated a clear association between advanced cervical changes and niche formation. Women with a niche had a significantly greater cervical dilatation (6.0 ± 2.3 cm vs. 4.1 ± 1.9 cm), effacement (58.2 ± 24.4% vs. 36.6 ± 24.4%), and advanced fetal station (0 or + 1 in 67.6% of niche cases) compared to those without niche (*p* < 0.001 for all). This suggests that the extensive cervical changes may increase mechanical stress on the lower segment, contributing to niche formation. By contrast, women with isolated myometrial defect exhibited lower cervical dilatation dilatation (3.6 ± 1.4 vs 5.3 ± 2.3), reduced effacement (29.4 ± 21.2 vs 50.2 ± 26.1) (*p* < 0.001 (3.6 ± 1.4 vs 5.3 ± 2.3), reduced effacement (29.4 ± 21.2 vs 50.2 ± 26.1) (*p* < 0.001 for both) and less fetal descent (−3 &−2 in 60%) (*p* = 0.005) compared to those without isolated myometrial defect. indicating that limited cervical remodeling may result in localized healing disturbances of the uterine wall. This distinction between niches & isolated myometrial defect represents an original contribution of the present study, as few previous reports analyzed them separately.

The correlation analysis demonstrated that both niche width and depth were positively associated with cervical dilatation(*r* = 0.591, 0.385; respectively), effacement (*r *= 0.547, 0.381; respectively), and fetal descent (*r *= 0.766, 0.545; respectively) (*p* < 0.05 for all), at the time of cesarean delivery, while residual myometrial thickness (RMT) showed a negative correlation with cervical dilatation (*r* = −0.368), effacement (*r* = −0.403), and fetal station (*r* = −0.381) (*p* < 0.05 for all). In contrast, for isolated myometrial defect dimensions revealed that defect width and depth were negatively associated with cervical dilatation (*r* = −0.447), effacement (*r* = −0.526), and fetal station (*r *= −0.491) at the time of cesarean delivery, whereas RMT demonstrated a positive correlation with these parameters. This suggests that more advanced cervical changes and greater fetal descent contribute to larger niche formation and thinner residual myometrium, potentially compromising scar integrity and increasing the risk of future complications such as uterine rupture or abnormal placentation. This indicates that cervical changes not only influence the occurrence but also the severity of scar abnormalities, Our findings extend previous observations by quantifying the strength of these correlations, offering a more analytical perspective. Unlike most prior studies that merely reported prevalence, our analysis quantified correlation strength, adding a novel analytical dimension. However, the cross-sectional nature limits inference about causality. On the other hand limited cervical remodeling and minimal fetal descent contribute to the development of smaller but structurally compromised scar defects, in which thicker residual myometrium may not guarantee functional scar integrity. This adds a new perspective to the literature, demonstrating that different scar abnormalities may have opposing pathophysiological mechanisms, underscoring the novelty of our findings.

The assessment of uterine findings revealed a mean total myometrial thickness of 10.9 ± 2.0 mm (range 6.0–17.0 mm). Despite this generally preserved thickness, localized scar abnormalities were observed in 37.0% (niches) and 27.2% (isolated myometrial defects) of cases, indicating that global myometrial integrity does not ensure proper healing. Instead, localized factors such as incision site, vascularity, and labor-related mechanical stress likely play pivotal roles. Cervical effacement and dilatation thin and shorten the lower uterine segment, predisposing to excessive stretching and niche formation [16], whereas limited cervical changes and high fetal station may lead to rigid uterine walls and suboptimal healing.

Beyond mechanical factors, impaired vascular integrity of the lower segment—supplied mainly by terminal branches with limited collateral flow—reduces oxygenation and tissue repair, explaining the vulnerability to delayed remodeling or rupture [17]. These findings have clinical relevance, as identifying women at risk for poor healing supports considering elective delivery once term is reached. Supporting studies include Elkashef et al. [12], who linked reduced scar–internal os distance to thinner residual myometrium (11.35 ± 3.32 mm), and *Vikhareva Osser & Valentine* [18] and *Kulshrestha *et al*.* [1], who reported that cervical dilatation > 5 cm, labor > 5 h, and low fetal station increased defect size. Consistently, *Lumbanraja *et al*. *[13]*, Handayani *et al*. *[19]*, Feldman *et al*. *[20]*, and Park *et al*. *[21] found that greater cervical dilatation, prolonged labor, and low fetal station predispose to niche formation. Conversely, *Jones *et al*. *[22]* and Rosli *et al*. *[23] observed that advanced cervical dilatation improves labor progression and reduces primary CS rates. Collectively, these data confirm the dual role of cervical changes—as both facilitators of vaginal birth and contributors to scar compromise when cesarean delivery becomes unavoidable, a concept that bridges physiological labor dynamics with postoperative uterine healing. This contextual understanding provides a basis for interpreting the contrasting evidence discussed below.

Nevertheless, contrasting evidence exists. *Pan *et al*.* [24], and *Dogru *et al*.* [9] found no significant association between cervical dilation and cesarean scar defect formation. While, *Dawood *et al*.* [25] reported that cervical dilatation during elective cesarean section is associated with thicker scars and a lower incidence of scar defects. These discrepancies emphasize the complex interplay of biological, surgical, and population-specific factors affecting scar remodeling and underline the importance of further standardized multi-centric studies.

Anatomical assessment revealed that niches were most frequent in isthmic scars, while isolated myometrial defects occurred predominantly in the uterine corpus. This pattern may reflect structural and vascular differences between the lower segment and corpus, as the lower uterine segment undergoes extensive remodeling and reduced perfusion during pregnancy and labor [26]. This finding aligns with *Meuleman *et al*.* [27], and *Dogru *et al*.* [9] who reported similar isthmic predominance. Recognizing these spatial variations can guide obstetric surgeons toward more precise incision placement and help identify patients who may benefit from tailored postoperative monitoring.

Overall, the present study refines the understanding of cesarean scar physiology by linking specific intrapartum parameters to measurable post-healing outcomes. These findings collectively reinforce the concept that cesarean timing and cervical status are not only procedural decisions but also key determinants of uterine healing quality, providing both scientific and clinical implications for optimizing obstetric care.

### Strengths

This study has several notable strengths. First, unlike most previous reports that evaluated cesarean scar abnormalities as a single entity, our work provided a distinct analysis of niches and isolated myometrial defects as separate entities. This distinction provided a clearer understanding of their potentially different pathophysiological mechanisms and healing patterns.

Second, by linking specific labor dynamics—particularly cervical dilatation, effacement, and fetal station—to the type and severity of scar abnormalities, our findings offer direct clinical implications. They highlight that while advanced cervical changes are typically associated with reduced cesarean section rates, when cesarean delivery becomes inevitable at such stages, it may result in more severe uterine wall damage. Therefore, close monitoring of labor progression and timely decision-making regarding cesarean delivery once indicated are crucial to minimize scar complications.

Third, from a patient-centered perspective, identifying women with large niches allows for targeted follow-up during the third trimester, enabling early anticipation and management of potential complications in subsequent pregnancies. This enhances the translational value of our findings, bridging the gap between ultrasound-based research and practical obstetric care.

Collectively, these strengths emphasize the originality, clinical applicability, and translational potential of the current study.

### Limitations

This study has several limitations. The sample size, although sufficient for initial observations, may limit generalizability to broader populations. Long-term follow-up to evaluate the impact of scar abnormalities on future pregnancies, including the risk of uterine rupture, abnormal placentation, and adverse perinatal outcomes, was not performed. Transvaginal ultrasound, while highly accurate for niche detection, is subject to interobserver variability, which may affect measurements. Additionally, the method of uterine closure and type of suture material were not standardized, although both are known to influence scar healing. The observational design of the study precludes establishing causal relationships. Therefore, our findings should be interpreted with caution, and further larger multi-centric longitudinal studies with long-term follow up are warranted. And also, Although 16 women delivered preterm, all cases were near-term (≥ 35 weeks). The absence of earlier preterm deliveries (< 34 weeks) limits the ability to assess potential differences in lower uterine segment development and scar formation in very preterm cases.

## Conclusion

This study provides valuable insights into cesarean scar topography, niche formation, and isolated myometrial defect in women undergoing their first cesarean section at different stages of 1 st stage of labor. The findings highlight that niche formation is significantly associated with more advanced cervical changes at the time of cesarean delivery, while myometrial defects are more frequently observed in cases with less cervical dilation and higher fetal station. These insights may support more individualized decision-making during labor and optimize timing of cesarean delivery to improve uterine healing outcomes [17].

## Data Availability

The datasets used and analyzed during the current study are available from the corresponding author on reasonable request.

## Abbreviations

AUB: Abnormal uterine bleeding
CBC: Complete blood count
CS: Cesarean section
EDHS: Egypt Demographic and Health Survey
RMT: Residual myometrial thickness
